# COVID-19, Neuropathology, and Aging: SARS-CoV-2 Neurological Infection, Mechanism, and Associated Complications

**DOI:** 10.3389/fnagi.2021.662786

**Published:** 2021-06-03

**Authors:** Rajkumar Singh Kalra, Jaspreet Kaur Dhanjal, Avtar Singh Meena, Vishal C. Kalel, Surya Dahiya, Birbal Singh, Saikat Dewanjee, Ramesh Kandimalla

**Affiliations:** ^1^AIST-INDIA DAILAB, National Institute of Advanced Industrial Science and Technology (AIST), Tsukuba, Japan; ^2^Department of Computational Biology, Indraprastha Institute of Information Technology Delhi, Okhla Industrial Estate, New Delhi, India; ^3^CSIR-Centre for Cellular and Molecular Biology (CCMB), Hyderabad, India; ^4^Department of Systems Biochemistry, Institute of Biochemistry and Pathobiochemistry, Faculty of Medicine, Ruhr-University Bochum, Bochum, Germany; ^5^Conservative Dentistry and Endodontics, Maharishi Markandeshwar College of Dental Sciences and Research, Ambala, India; ^6^ICAR-Indian Veterinary Research Institute (IVRI), Regional Station, Palampur, India; ^7^Advanced Pharmacognosy Research Laboratory, Department of Pharmaceutical Technology, Jadavpur University, Kolkata, India; ^8^Applied Biology, CSIR-Indian Institute of Chemical Technology (IICT), Hyderabad, India; ^9^Department of Biochemistry, Kakatiya Medical College, Warangal, India

**Keywords:** COVID-19, SARS-CoV-2, neuropathology, aging, neuroinvasion, neuroinfection, pandemic, neurodegenerative disease

## Abstract

The spectrum of health complications instigated by coronavirus disease 2019 (COVID-19, caused by the novel severe acute respiratory syndrome coronavirus 2 or SARS-CoV-2) pandemic has been diverse and complex. Besides the evident pulmonary and cardiovascular threats, accumulating clinical data points to several neurological complications, which are more common in elderly COVID-19 patients. Recent pieces of evidence have marked events of neuro infection and neuroinvasion, producing several neurological complications in COVID-19 patients; however, a systematic understanding of neuro-pathophysiology and manifested neurological complications, more specifically in elderly COVID-19 patients is largely elusive. Since the elderly population gradually develops neurological disorders with aging, COVID-19 inevitably poses a higher risk of neurological manifestations to the aged patients. In this report, we reviewed SARS-CoV-2 infection and its role in neurological manifestations with an emphasis on the elderly population. We reviewed neuropathological events including neuroinfection, neuroinvasion, and their underlying mechanisms affecting neuromuscular, central- and peripheral- nervous systems. We further assessed the imminent neurological challenges in the COVID-19 exposed population, post-SARS-CoV-2-infection. Given the present state of clinical preparedness, the emerging role of AI and machine learning was also discussed concerning COVID-19 diagnostics and its management. Taken together, the present review summarizes neurological outcomes of SARS-CoV-2 infection and associated complications, specifically in elderly patients, and underlines the need for their clinical management in advance.

## Introduction

Primary physio-pathological evidence of Coronavirus Disease 2019 (COVID-19) exhibited severe respiratory and cardiovascular complications in the novel severe acute respiratory syndrome coronavirus 2 (SARS-CoV-2) infected patients. Emerging reports lately revealed that SARS-CoV-2 infection develops a range of neurological complications (COVIDview, 2020). These complications are produced either by the direct contact of SARS-CoV-2 with the nervous system or by an indirect impact of immune-response during- or post-infection (Ellul et al., 2020). As an estimate, ~35.6% of total COVID-19 cases were found to exhibit multiple neurologic manifestations (Tsai et al., 2020). Although neurological consequences of these events are evident across all age groups, yet, elderly COVID-19 patients remain at remarkably high risk (COVIDview, 2020). An acute phase of direct SARS-CoV-2 infection could produce immediate neurological complications, however, a secondary phase might take months to surface after the infection (Beghi et al., 2020). Multiple nervous tissue/cell types including macrophages, microglia, or astrocytes are invaded by coronaviruses that can cause direct damage to the nerves (Beghi et al., 2020; Wu et al., 2020). Recent reports also underlined evidence of neurotoxicity primarily caused by immune injury, hypoxia-induced injury, and angiotensin-converting enzyme 2 (ACE2, a SARS-CoV-2 host receptor) binding (Beghi et al., 2020; Wu et al., 2020). A recent systematic review analyzed the neuropathological features in patients who have died post-SARS-CoV-2 infection and it revealed that the majority of these patients were elderly (*n* = 66, 45%) and males (*n* = 79, 54%; Pajo et al., 2021). The striking neuropathological features they exhibited include diffuse edema (17%), gliosis (having diffuse microglia and astrocytes activation, 35.6%), cortical and subcortical regional infarctions in the brain (2.7%), intracranial (subarachnoid and punctate) hemorrhage (12.4%), arteriosclerosis (29.5%), hypoxic-ischemic injury (28.1%), and inflammation (35.6%). These observed features were suggested to be caused by direct cytopathic and indirect effects derived from host-specific inflammatory response post-SARS-CoV-2 infection (Pajo et al., 2021). These events greatly contribute to the development of neuro-pathophysiological symptoms in elderly COVID-19 patients. Although the long–term neurological complications in individuals who had COVID-19 are still unknown, similar viral infections were shown to exhibit neurological complications after months or years of infection by developing neuropsychiatric and cognitive impairment (Troyer et al., 2020).

The olfactory tract is a preferred route of coronavirus infection to the brain at an early stage, whereas evidence of brain invasion through systemic circulation is scarce (Wu et al., 2020). The common neurological complications resulting from direct infection are found to be encephalitis, myelitis, meningitis, and inflammatory central nervous system (CNS) vasculitis; whereas, immune-related CNS, peripheral nervous system (PNS) diseases, and the Guillain-Barré syndrome (GBS) emerged as the major post-infection complications (Beghi et al., 2020; Ellul et al., 2020). By an estimate, 20% of the COVID-19 patients with ICU admittance had neurological complications and faced a high risk of mortality (Fotuhi et al., 2020). Of note, in elderly patients, SARS-CoV-2 instigated neurologic and immunologic complications that have produced severe consequences leading to neurodegenerative diseases (Lennon, 2020; Pavel et al., 2020). Taken together, in the present report we comprehensively reviewed the SARS-CoV-2 routes, neuro-infection or -invasion mechanism(s), their emergent and post-infection neurological manifestations, with a special focus on the elderly patients. We have also shed light on the emerging artificial intelligence (AI) and machine learning diagnostic applications for COVID-19 patients.

## SARS-COV-2 Manifested Neurological Complications

**Figure 1:**
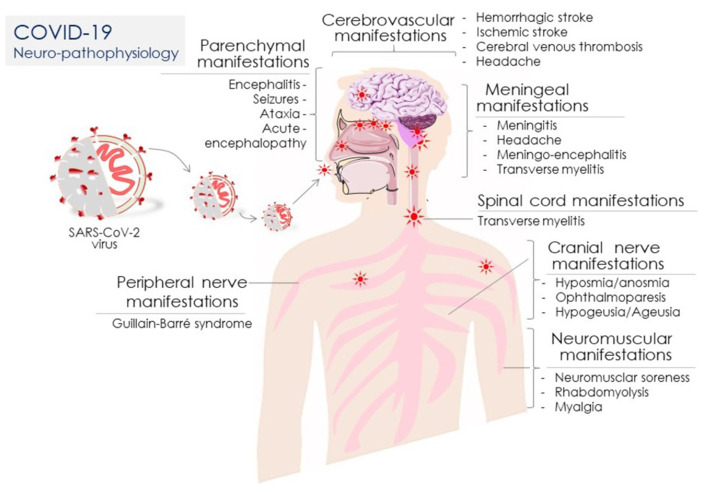
Coronavirus disease 2019 (COVID-19) neuro-pathophysiology: COVID-19 clinical manifestations associated with diverse neuronal systems/organs including the peripheral nerve, parenchymal, cerebrovascular, meningeal, spinal cord, neuromuscular, and cranial nerve in SARS-CoV-2-infected patients.

An early clinical case series from Wuhan, China revealed a significant relevance of SARS-CoV-2 infection with developing neurologic complications (Mao et al., 2020). It was estimated that out of 214 COVID-19 patients, 36.4% developed neurologic complications including CNS manifestations (dizziness, headache, acute cerebrovascular disease, diminished consciousness, ataxia, and seizures), PNS manifestations (sensory ailments and neuralgia), and neuromuscular injury (Mao et al., 2020; Figure 1). A retrospective report from Wuhan showed that 5% of a total of 221 COVID-19 patients had incidences of acute ischemic stroke (Guan et al., 2020). A similar retrospective report from Wuhan revealed that 20% of 113 COVID-19 patients suffered from hypoxic encephalopathy (Chen et al., 2020a).

To assess neurological complications in elderly COVID-19 patients, a cross-hospital nationwide investigation in the UK comprising 125 COVID-19 patients (avg. age 71 years) analyzed clinical data for neurological and psychiatric manifestations and revealed that 62% of the patients suffered from cerebrovascular events, among which 74% were reported with ischemic stroke, 23% developed unspecified encephalopathy and 1% acquired CNS vasculitis (Varatharaj et al., 2020). Noticeably, among the total patients, 31% developed altered mental complications—encephalitis (18%) and intracerebral hemorrhage (12%; Varatharaj et al., 2020). The remaining 59% of the patients met the clinical case definitions of psychiatric diagnoses, among which 43% possessed new-onset psychosis, 26% acquired neurocognitive syndrome, and 17% exhibited an affective disorder (Varatharaj et al., 2020). Of note, 82% of total enrolled COVID-19 patients having cerebrovascular events were aged more than 60 years, which is suggesting that elderly patients are at high risk for COVID-19 associated neurological complications advancing to greater lethality. A retrospective meta-analysis enrolling 1,558 COVID-19 patients from a total of six studies revealed that cerebrovascular disease is a potential risk factor (Wang et al., 2020a). A multi-centric report involving 184 COVID-19 patients admitted to ICU in three Dutch hospitals showed a considerably high (31%) risk of thrombotic complications, while the death of 23 patients among these underlined the severity of such complications (Klok et al., 2020). A multi-centric retrospective study from Chicago, USA, further corroborated the fact that neurological manifestation is a major risk factor in hospitalized COVID-19 patients (Liotta et al., 2020). In this study, among the 509 COVID-19 patients, neurological manifestations were revealed at onset (42.2%), hospitalization (62.7%), and other stages of COVID-19 disease (82.3%; Liotta et al., 2020). Another multi-centric retrospective study of SARS-CoV-2 infected hospitalized patients in New York City assessed the prevalence of neurologic disorders, also it analyzed in-hospital mortality and compared manifested features between COVID-19 patients with and without neurologic disorders (Frontera et al., 2021). It strikingly revealed that 13.5% of patients developed a new neurologic disorder in 2 days median time from the onset of COVID-19 symptoms. It included toxic/metabolic encephalopathy (6.8%), stroke (1.9%), seizure (1.6%), and hypoxic/ischemic injuries (1.4%), though no patient had meningitis/encephalitis or myelopathy/myelitis due to SARS-CoV-2 infection (Frontera et al., 2021). Of note, neurologic disorders were more common in aged patients, wherein the male, white, diabetic, hypertensive, intubated population was vulnerable (all *p* < 0.05) and faced an increased risk of in-hospital mortality and lesser recovery. This survey suggested that observed neurologic features may be sequelae of severe systemic illness (Frontera et al., 2021).

The neurologic manifestations i.e., myalgias, headaches, dizziness, dysgeusia, and anosmia, of all encephalopathies were found to be associated with poor health outcomes in admitted patients, irrespective of COVID-19 severity (Liotta et al., 2020; Figure 1). In the line, a 2-centric retrospective study from Spain also affirmed that neurological manifestations were frequent in admitted 841 COVID-19 patients (Romero-Sanchez et al., 2020). Montalvan et al. (2020) confirmed it further by systematically reviewing a total of 67 studies, wherein they found that risk of encephalitis, neuropathy, demyelination, and stroke are associated with COVID-19 (Montalvan et al., 2020). Providing insights on the SARS-CoV-2 infection route to the nerve tissue, it was revealed that the virus invasion occurs through the lamina cribrosa or olfactory tract and disperses through the trans-synaptic transfer (Montalvan et al., 2020). Furthermore, another systematic review assessed a greater risk of secondary neurologic complications in hospitalized COVID-19 patients (Herman et al., 2020); while, another estimate claimed that 1 out of 3 COVID-19 patients could acquire an altered mental state (Belluck, 2020).

**Figure 2:**
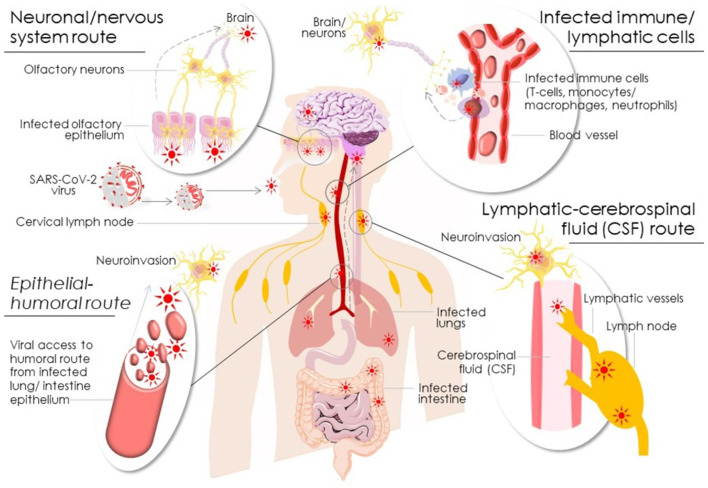
Schematic diagram showing potential modes of SARS-CoV-2 neuroinfection *via* neuronal/nervous, epithelial-humoral, infected immune/lymphatic, and lymphatic-cerebrospinal fluid routes.

SARS-CoV-2 manifested complications that include the CNS, PNS, and neuromuscular system, range from mild to severe, and can also appear in patients with asymptomatic SARS-CoV-2 infection. SARS-CoV-2 infects the host by establishing the binding of its spike (S) glycoproteins to the host ACE2 receptor that expresses in the brain, gastrointestinal tract (GI), respiratory tract, lung parenchyma, and endothelial cells, and therefore it serves as a potential target for the direct COVID-19 inhibitory regimens (Kalra et al., 2021) or indirect suppressive strategies including miRNAs (Li et al., 2018). The most common symptoms associated with SARS-CoV-2 are dry cough, fever, and lethargy; however, aged adults are more susceptible to severe infection involving shortness of breath, pneumonia, and acute respiratory distress syndrome (ARDS) leading to higher mortality incidence (Table 1). A natural decline in ACE2 levels, elevated angiotensin signaling, and subsequently chronic low-grade inflammation that develops with advanced age, termed inflammaging, might contribute to the severity and comorbid diabetic and cardiovascular complications in aged individuals (Alghatrif et al., 2020; Kalra et al., 2020). SARS-CoV-2 is a neuro-invasive and neurotrophic virus. Studies implicated that neurological manifestations are primarily associated with the severity of SARS-CoV-2 infection, which involves loss of taste, smell, consciousness, vision, seizures, neuralgia, and lack of coordination (Mao et al., 2020). SARS-CoV-2 can enter the CNS through the olfactory lobe and hematogenous route (Figure 2). A gradual decline of the blood-brain barrier (BBB) is associated with normal aging, which may enhance the effect of SARS-CoV-2 in aged individuals (Montagne et al., 2015). SARS-CoV-2 causes neurodegeneration, demyelination, and cellular senescence upon entry; all of these potentiate brain aging and aggravate the underlying pathophysiology of neurodegeneration (Lennon, 2020; Montalvan et al., 2020; Palao et al., 2020; Pavel et al., 2020).

**Table 1:** Summary of common peripheral nervous system (PNS), central nervous system (CNS), cerebrovascular, and intracerebral neurological complications in elderly Coronavirus disease 2019 (COVID-19) patients.

Neurological complications	Manifestations	Investigation/Region/ Study type	Clinical features/Symptoms	COVID-19 Diagnostics	Neurological investigation (CSF scans, neuroimaging, neurophysiology)
CNS disease	Encephalitis	Sohal and Mansur (2020); 1 case, USA, Case report.	72-year-old male patient. Weakness and lightheadedness. Altered mental status; Seizures (On day 2 post-hospitalization).	RT-PCR + ve	Head CT: no acute changes. 24-h EEG: six left temporal seizures and left temporal sharp waves that were epileptogenic.
		Paniz-Mondolfi et al. (2020); 1 case, USA, Case report.	74-year-old male patient. History of Parkinson’s disease. Fever, confusion, and agitation.	RT-PCR + ve (nasopharyngeal)	Head CT: no acute changes.
		Zhou et al. (2020b); 1 case, China, Case report.	56-year-old patient. SARS-CoV-2 infection and pneumonia.	SARS-CoV2 + ve (CSF sequencing)	NR
	Acute disseminated encephalomyelitis	Zanin et al. (2020); 1 case, Italy, Case report.	54-year-old female patient. Agitation, decreased consciousness, and seizures after many days of ageusia and anosmia.	RT-PCR + ve	CSF: normal; Brain and spine MRI: periventricular confluent white matter lesions. Numerous cord lesions from (bulbomedullary junction to T6 level).
	Myelitis	Zhao et al. (2020b); 1 case, China, Case report.	66-year-old male patient. Fever, dyspnoea, and asthma. Developed acute flaccid paralysis of lower limbs (5 days after the beginning of respiratory symptom). Urinary and fecal incontinence. Sensory level at T10.	RT-PCR + ve (nasopharyngeal)	Brain CT: lacunar infarcts; spinal imaging not done
PNS disease	Guillain-Barré syndrome	Camdessanche et al. (2020); 1 case, France, Case report.	64-year-old male patient. Developed paraesthesia and progressive weakness in all limbs. Areflexia and loss of vibration sense. Later developed dysphagia and respiratory insufficiency.	RT-PCR + ve (nasopharyngeal)	CSF: normal. Nerve conduction and electromyography: acute inflammatory demyelinating polyneuropathy.
		Zhao et al. (2020b); 1 case, China, Case report.	61-year-old female patient. Progressive weakness of limbs and severe fatigue. Areflexia in lower limbs and reduced sensation distally. Dry cough and fever (after 7 days).	RT-PCR + ve (oropharyngeal)	CSF: normal. Nerve conduction study: acute inflammatory demyelinating polyneuropathy.
GBS variants and other neuropathies	Miller-Fisher Syndrome	Gutierrez-Ortiz et al. (2020); 1 case, Spain, Case report.	64-year-old male patient. Cough, fever, malaise, anosmia, headache, and ageusia. Developed right inter-nuclear opthalmoparesis with right fascicular oculomotor palsy, ataxia, and areflexia.	RT-PCR + ve(oropharyngeal)	CSF: normal. Brain CT with contrast: normal.
	Ophthalmoplegia	Dinkin et al. (2020); 1 in USA, Case report.	71-year-old female patient. Had isolated ophthalmoplegia (post-few days of cough and fever; right abducens palsy).	RT-PCR + ve (nasal)	CSF: normal opening pressure; brain MRI: enhancement of the optic nerve sheaths and posterior Tenon capsules.
	Rhabdomyolysis	Jin and Tong (2020); 1 case of rhabdomyolysis, China, Case report.	60-year-old male patient. Weakness and tenderness in lower limbs (15 days after beginning of fever and cough).	RT-PCR + ve (throat swab)	NR
Cerebrovascular disease	Ischaemic stroke	Avula et al. (2020); 4cases, USA, Case series.	4 patients (73–88 years old). Had hypertension; 3 had dyslipidaemia, 1 diabetes and neuropathy. 3 patients exhibited acute new focal neurological deficit and 1 showed altered mental status.	All RT-PCR + ve	All 4 had unifocal infarcts: 3 on CT, 1 on brain MRI.
		Beyrouti et al. (2020); 6 cases, UK, Case report.	6 patients (53–83 years old. 5 male and 1 female). 3 had hypertension, 2 ischemic heart disease, 2 atrial fibrillations, 1 had previous stroke, and 1 was a heavy smoker and alcohol drinker. All had respiratory symptoms (at avg. 13 days) before or after neurological symptom onset.	All RT-PCR + ve	Scans (CT and brain MRI) showed unifocal infarcts in 4 patients. 1 had bilateral infarcts on a follow-up brain MRI; 2 had bilateral infarcts on initial scans.
		Li et al. (2020a); 11 cases, China, Single-center, retrospective study.	11 patients (57–91 years old; 6 female and 5 male). 9 had hypertension, 6 diabetes, 3 cardiovascular disease. All had respiratory symptoms (at avg. 11 days) before neurological symptoms onset.	All RT-PCR + ve	NR
		Morassi et al. (2020); 4 cases, Italy, Case series.	4 patients (64–82 years old). 3 had hypertension, 2 had a previous stroke or transient ischemic condition and aortic valve disease, and 1 was a smoker with a previous myocardial infarction. 3 developed neurological manifestations during hospitalization, 1 exhibited episodes of transient loss of consciousness.	All RT-PCR + ve (nasopharyngeal)	1 patient had CSF: normal leukocyte count, protein, and IgG index. All had multifocal infarcts on brain CT or MRI; the patient presenting with transient loss of consciousness and ensuing confusion.
	Intracerebral hemorrhage	Morassi et al. (2020); 2 cases, Italy, Case series.	2 patients (57 years old). Admitted to hospital with critical COVID-19 condition; (at 14 and 17 days after onset of cough and fever), they had bilaterally fixed dilated pupils and coma (GCS 3/15).	Both RT-PCR + ve (nasopharyngeal)	1 patient had bilateral cerebellar hemorrhages on brain CT with hydrocephalus; the other had a large frontal hemorrhage with displaced ventricles and multiple smaller hemorrhages.

At the beginning stage of infection, patients with the COVID-19 focus on managing cough, dyspnea, fever, and breathing complications. However, it is evident from studies that it gradually led to an increase in neurological complications, such as stroke, seizures, anosmia, encephalopathy, and paralysis (Li et al., 2020b; Mao et al., 2020). In 2002 and 2013, during the epidemics of SARS-CoV-1 and Middle East Respiratory Virus (MERS) respectively, the virus caused a detrimental effect in multiple organs, including the brains, nerves, and neuromuscular tissues. SARS-CoV-2, shares homology with SARS-CoV-1 and MERS and therefore emerges as an essential player in causing CNS and PNS injury, either direct or indirect (Nath, 2020; Wu et al., 2020). Neurological abnormalities have been documented in the patients who required hospitalization for COVID-19, respiratory illness, and acute respiratory distress syndrome (ARDS; Helms et al., 2020; Mao et al., 2020). In a clinical case series, neurological symptoms are restricted to general conditions such as headache, loss of smell and taste, dizziness, and malaise in mild conditions, which are routinely observed with viral infection (Mao et al., 2020). Among 1,420 mild-to-moderately infected COVID-19 patients, 70% of the patients experienced headache, which is a prominent neurological manifestation. Noticeably, severe neurological complications can be seen in mild-infected COVID-19 patients in the multiple clinical reports, while patients with pre-existing comorbidities had severe complications resulting in significantly high mortality (Iadecola et al., 2020; Merkler et al., 2020; Oxley et al., 2020; Yaghi et al., 2020; Table 1).

### Central Nervous System (CNS) Manifestations

Symptoms related to mental status such as confusion, tiredness, and agitation, collectively known as encephalopathy, have been described in the COVID-19 in few clinical reports (Table 1). Diagnostic criteria for detecting encephalitis have been established and include fever, seizures, focal brain abnormalities, disturbed mental status, and white blood cells in the lymphatic-cerebrospinal fluid (CSF; Venkatesan et al., 2013). In the clinical case series, the cognitive level was primarily affected in most critically ill COVID-19 patients with ARDS (Helms et al., 2020) compared to COVID-19 patients with only respiratory illness (Mao et al., 2020). It is unclear whether the alteration in mental status is due to encephalitis caused by systemic disease or directly caused by SARS-CoV-2 infection. However, several reports suggest that COVID-19 patients exhibited well-established diagnostic markers for encephalitis (Efe et al., 2020; Farhadian et al., 2020; Huang et al., 2020a; Pilotto et al., 2021). In the very first reported case of meningitis/encephalitis associated with COVID-19, Moriguchi et al. (2020) observed SARS-CoV-2 level in the CSF but found only a modest amount of viral RNA load. In another case study, a biopsy from COVID-19 patient showed neuronal loss due to hypoxia and perivascular lymphocyte infiltration confirming temporal lobe encephalitis (Efe et al., 2020). However, Efe et al. (2020) did not detect SARS-CoV-2 in the brain or CSF. Brain tissue samples from autopsies of COVID-19 patients negative for evidence of encephalitis and CSF samples from COVID-19 patients with neurological abnormalities have not revealed evidence of SARS-CoV-2 (Kandemirli et al., 2020). Other significant mental status indicators such as confusion, delirium, and coma may be common symptoms in COVID-19 patients. These indicators are frequently associated with hypotension, kidney disease, usage of sedatives, hypoxia, and prolonged bedridden and isolation conditions—all these factors are significant contributors to the progression of encephalopathy (Helms et al., 2020; Mao et al., 2020; Martin-Jimenez et al., 2020; Maas et al., 2021; Rogers et al., 2021). Although SARS-CoV-2 affects all ages, adults aged 65 and older are at higher risk of severe disease, hospitalization, ICU use, and death. Geriatric age patients are prone to loss of consciousness, disorientation, and other cognitive disturbances. Delirium is a common symptom of older people with COVID-19 during hospitalization. Despite the lack of clinical data and histopathological evidence of encephalitis and occurrence of other alternative events impacting mental status, these data so far hint that the potential invasion of SARS-CoV-2 in the brain may be the sporadic cause of encephalopathy.

In retrospective clinical studies, 11 patients out of 221 showed acute ischemic stroke, along with one patient who developed cerebral venous thrombosis and cerebral hemorrhage. The majority of these patients were elderly and were suffering from severe COVID-19 along with common comorbidities (Li et al., 2020b). A small clinical case study from the UK, comprising six severely affected patients showed cerebral infarcts and elevated D-dimer levels, suggesting a coagulopathy (Beyrouti et al., 2020). In another small case study of five young patients, COVID-19 related strokes were shown to cause large-vessel infarct (Oxley et al., 2020). Al Saiegh et al. (2020) in a small case report, could not demonstrate the presence of SARS-CoV-2 in the CSF of aged patients that had an ischaemic stroke. Hypoxia, produced by lack of oxygen, increases stroke incidence due to impairment in sleep structure, increases blood pressure, atherosclerosis, promotes micro thrombosis, and decreases the blood flow (Lee et al., 2019). In a case series, Mao et al. (2020) showed that six hospitalized COVID-19 patients exhibited acute cerebrovascular disease, among these, five had a severe infection (5/88) and one was in non-severe condition (1/125). The symptoms of hypoxia are coupled with COVID-19, and it was predicted to be a consequence of S-protein’s interaction with hemoglobin (Agrawal et al., 2021); therefore, it was believed to potentiate the patient’s necessity for ventilator support. Of note, in a single-centric case series, the COVID-19 patients admitted to ICU were relatively old, and they had a more significant number of comorbid conditions, such as diabetes, cardiovascular complication, hypertension, and cerebrovascular disease, in comparison to those who did not require ICU (Wang et al., 2020b). The findings of this clinical case series imply that aging and comorbidities may be risk factors for poor neurological and survival outcomes (Wang et al., 2020a; Table 1). Seizure is a rare symptom in the COVID-19 setting. Reports involving a retrospective multi-centric study and a case series could not observe stroke in COVID-19 patients despite metabolic alteration (Lu et al., 2020; Mao et al., 2020).

### Peripheral Nervous System (PNS) Manifestations

Moein et al. (2020) in the earliest analysis derived from a cohort of 100 COVID-19 patients showed that loss of smell sensation (anosmia) and taste sensation (ageusia) is the most common neurological manifestation of COVID-19, even in mild to moderate cases. Smell sensation is more affected compared to the taste sensation. Emerging evidence suggests that SARS-CoV-2 can enter the neuronal cells through the olfactory nerve and spreads to the olfactory bulb (Figure 2). Lechien and colleagues in a multi-centric European study and a case report showed that 86% and 88% of COVID-19 patients respectively, reported the loss of smell and taste (Lechien et al., 2020a, b). Cranial neuropathy is an erratic event in the setting of COVID-19; however, one case study involving a 40-year-old female COVID-19 patient showed isolated oculomotor nerve palsy in severely ill patients, which could be due to inflammatory reaction against SARS-CoV-2 (Wei et al., 2020). COVID-19 can cause detrimental effects to the peripheral nerves, cranial nerve, and neuromuscular tissue. Dyspnea, facial weakness, inability to stand or walk, or struggling with weaning off respiratory ventilators might be partially due to GBS expedited by COVID-19. GBS is frequently observed neurological complications in COVID-19 (Zhao et al., 2020a). Miller-Fisher syndrome is measured by the acute onset of external loss of tendon reflexes, ataxia, and ophthalmoplegia. In a clinical case report in Spain, involving a hospitalized 64-year old male COVID-19 patient, clinical features and eye movement abnormalities were found to be consistent with the diagnosis of Miller-Fisher Syndrome and polyneuritis cranialis. The symptoms consisted of ataxia, fascicular palsy, areflexia, anosmia, and ageusia (Table 1). This patient received Intravenous immunoglobulin (IVIg) and showed rapid recovery (Gutierrez-Ortiz et al., 2020). In a case report from three hospitals in northern Italy, comprising five patients who had GBS syndrome, one patient diagnosed with COVID-19 exhibited sensory ataxia, facial weakness, and facial nerve, though this patient responded positively to the treatment with IVIg and improved within a week (Toscano et al., 2020). The other four patients showed more GBS and a variable degree of COVID-19 symptoms. The severity and mortality of COVID-19 patients depend on age and pre-existing comorbidities, and ongoing treatment regimen. Multiple sclerosis (MS) is particularly prevalent in young adults; however, a substantial number of individuals with MS are older than 60 years (Minden et al., 2004). Managing MS during the COVID-19 pandemic is critical for patient’s health management as there are no evidence-based guidelines and published literature yet available. In general, elderly patients (≥65 years) are susceptible to COVID-19 severity and mortality. Analyses from a Sonya Slifka Longitudinal MS study indicated that 10–20% of MS patients are more than 65 years old (Minden et al., 2004). However, whether it could impact the COVID-19 mortality in older patients given their impaired immune regulation, yet need to be elucidated (Berger et al., 2020). Besides the age, other comorbidities are correlated in the MS population cohorts as well with increased risk of severity and mortality (reviewed by Marrie et al., 2015a, b).

In one cross-sectional study in Europe, 1,931 MS patients were engaged to determine the mortality associated with COVID-19 (Bsteh et al., 2020). Out of 1,931 patients, 63% showed a low risk of COVID-19 mortality, 26% had mild risk, 8.8% had moderate risk, while 0.9% exhibited a high risk of COVID-19 mortality. Only one patient received disease-modifying treatment (DMT) in the high-risk category, and none had any immunosuppressive therapy. The increased risk of COVID-19 mortality is below 1% in the population-based MS cohort (Bsteh et al., 2020). At the beginning of the COVID-19 pandemic, clinicians recommended delaying treatment with DMT in MS patients (Giovannoni et al., 2020). Recent data has suggested that COVID-19 positive MS patients are not different from the general MS population (Parrotta et al., 2020). However, clinicians and physicians need to be vigilant for prescribing the drugs and recommendations regarding MS to guide their patients during the COVID-19 pandemic.

### Neuromuscular Dysfunction/Injury

As per a clinical case series analysis of 214 COVID-19 patients, 11% of patients were reported to have evidence of neuromuscular injury (Mao et al., 2020). The damage was more prominent in severely affected (19%) than non-severely affected individuals (5%); however, these results do not indicate whether the damage is due to the COVID-19 neuromuscular infection. Such injuries were suggested to be due to SARS-CoV-2 infection-mediated release of pro-inflammatory cytokines. However, no clinical details are yet available beyond the presence of neuromuscular pain. Lately, two reports suggested it to be rhabdomyolysis as its clinical features were manifested in COVID-19 infected patients (Jin and Tong, 2020; Suwanwongse and Shabarek, 2020). Rhabdomyolysis is skeletal/neuromuscular damage that can be a manifestation of COVID-19. In a case report, a 35-year-old female was found to have rhabdomyolysis correlated with COVID-19 (Alrubaye and Choudhury, 2020). Clinical data from the report suggests that clinicians should examine the level of liver enzyme and myalgia, which could serve as clinical features of rhabdomyolysis in COVID-19 patients. Detailed analysis of the CSF pro-inflammatory and T-cell response to SARS-CoV-2 is urgently warranted to comprehensively understand the neuromuscular manifestation in COVID-19 patients.

Although we presently lack a distinct and detailed analysis of SARS-CoV-2 -manifested neurological complications including CNS, PNS, and neuromuscular injuries in the aged population, we summarize the key clinical reports/case studies involving elderly COVID-19 patients in Table 1.

## Post COVID-19-Infection Neurological Complications: What Is Known So Far

Like COVID-19, other coronaviruses *viz*. SARS-CoV-1 and Middle East respiratory syndrome (MERS-CoV) have also been associated with various prolonged neurological complications (Chan et al., 2003; Lee et al., 2018). As discussed earlier, the most common neurological difficulties in COVID-19 include anosmia, ageusia, and headache, moreover, more serious complications, such as stroke, impaired consciousness, seizures, and encephalopathy have also been reported. Reports on these neurological and neuropsychological complications during and after the course of COVID-19 infection are growing rapidly. Focusing on the aging population, we have here limited our discussion about post-COVID-19 neurological and neuropsychological complications reported only in elderly patients.

Acute disseminated encephalomyelitis (ADEM), an autoimmune disease of the CNS, that mainly affects children has been observed in SARS-CoV-2 infected patients. However, most of the cases reported to be diagnosed with ADEM post-COVID-19 have been aged 50 years or above. But this might be biased given the higher prevalence of COVID-19 in adults. Amongst these reported cases was a 51-year-old female who developed clinical coma and an impaired oculocephalic response to one side post-COVID-19 infection, which was later diagnosed to be acute multifocal demyelinating lesions (Parsons et al., 2020). Another case study reported a 64-year-old woman with ADEM, who was hospitalized with mild behavioral abnormalities, headache, bilateral relative afferent pupillary defect, ageusia and anosmia, severe visual loss, right abdominal sensory level, and left-sided lower limb hyperreflexia with the Babinski sign (Novi et al., 2020). Both these patients recovered with the administration of high-dose steroids and intravenous immunoglobulins. ADEM was also diagnosed in the post-mortem biopsy of a 71-year-old male (Reichard et al., 2020). In another case study, similar immune-mediated brain damage was also detected in a 58-year-old male patient. The patient was hospitalized with low consciousness and loss of ability to walk. The patient was tested positive for SARS-CoV-2 infection even though pulmonary symptoms like cough or dyspnea were not observed. Though the patient initially responded to steroids but then died of status epilepticus (Abdi et al., 2020). Another case study of a likewise neurological syndrome that commonly called as acute myelitis has also been reported in a 69-year-old female (Sotoca and Rodriguez-Alvarez, 2020). The first clinical case of COVID-19 associated acute necrotizing hemorrhagic encephalopathy was reported by Poyiadji et al. (2020) in a 58-year-old female airline worker. This rare neurological condition was attributed to intracranial cytokine storm and disruption of the BBB without the direct viral invasion (Serrano-Castro et al., 2020). Investigating the case study of a 75-year-old man, Hayashi et al. (2020) have suggested that mild encephalitis/encephalopathy with reversible splenial lesion can also be considered as a neurological symptom in patients developing transient cerebellar ataxia or disorientations. To add to this list of neurological complications, vasculitis of CNS has also been reported to occur in a 65-year-old man (Hanafi et al., 2020). Along with CNS, the PNS has also been affected in COVID-19 patients. Pascual-Goni et al. (2020) reported a 60-year-old female patient who after 10 days of fever, hyposmia, nausea, and coughing experienced diplopia and right hemicranial headache that was later diagnosed as right abducens nerve palsy.

In terms of post-COVID-19 neuropsychological impact, a study concerning 700 clinically stable COVID-19 patients (mean age 50.2 ± 12.9 years) were examined for post-traumatic stress symptoms, and nearly 96% of the patient were found to be suffering from significant post-traumatic stress (Bo et al., 2020). A much larger cohort study involving 112 hospitalized patients and 2,001 non-hospitalized patients from Belgium and Netherland has revealed that symptoms like neuromuscular pain, dizziness, headaches, fatigue, and anosmia prevail also in asymptomatic or very mildly symptomatic patients even after months of contracting the disease (Goertz et al., 2020). Some patients treated for severe COVID-19 have been reported with disabling fatigue and impaired cognitive abilities after being discharged from the hospitals (Zhou et al., 2020a; Halpin et al., 2021). Also, the detection of delirium, the most common acute neuropsychiatric syndrome, has been significantly linked to COVID-19 in older adults and those with dementia (Poloni et al., 2020).

## Understanding The SARS-COV-2 Infection and Its Routes

Considering these features of neuropathological manifestations discussed above, here we shed light on the SARS-CoV-2 infection, its diverse routes, the mechanism(s), and associated neurological complications.

## Potential SARS-COV-2 Infection Routes

The neurotropic, neuroinvasive, and neurovirulent characteristics of SARS-CoV-2 were recognized in both humans and animals (Lima et al., 2020). Recent evidence suggested that coronaviruses can infect primary human neural cells, microglia, astrocytes, and oligodendrocytes (Lima et al., 2020). SARS-CoV-2 interacts with the host ACE2 receptor through the receptor-binding domain (RBD) of its Spike (S) protein. ACE2 receptor ubiquitously expresses in all human tissues including CNS and the endothelial cells. Emerging evidence reveals that SARS-CoV-2 binds to the ACE2 receptor to invade neurons in CNS *via* distinct routes as discussed here (Figure 2).

### Epithelial-Humoral Route

Coronaviruses can efficiently invade the epithelial-humoral route and disrupt the primary epithelial barrier to attain access into the bloodstream. ACE2 abundantly expresses on the alveolar epithelial cells (Type II) that makes these cells a preferred target for SARS-CoV-2 infection (Lima et al., 2020). Also, an abundant expression of ACE2 on the epithelial cells of the gastrointestinal tract raises their vulnerability for SARS-CoV-2 infection and access to the bloodstream (Li et al., 2020b; Figure 2). On ensuring access to the systemic circulation, SARS-CoV-2 disrupts the endothelial barrier of the BBB or the blood-cerebrospinal fluid barrier (BCSFB) *via* its interaction with ACE2 receptors at the endothelial cells and subsequent CNS contact (Li et al., 2020b). The presence of SARS-CoV-2 like particles in the neural and frontal lobe capillary endothelial cells of the patient who died with COVID-19 affirmed the hematogenous-endothelial route of SARS-CoV-2 neuroinvasion (Paniz-Mondolfi et al., 2020). Moreover, SARS-CoV-2 was also suggested to cross BBB by inducing inflammation or hypoxemia by stimulating the release of pro-inflammatory cytokines and chemokines (Li et al., 2020b; Lima et al., 2020). The pro-inflammatory cytokines *viz*. interferon-gamma (IFN-γ), interleukin (IL)-2, IL-6, IL-8, and tumor necrosis factor-alpha (TNF-α) were suggested to play a part in SARS-CoV-2 cellular invasion (Achar and Ghosh, 2020). However, it is unclear if induction of these pro-inflammatory cytokines is a result of SARS-CoV-2 cellular invasion activity or it reflects an elicited antiviral immune response for its neutralization/clearance. Multiple clinical reports exhibited a surge in IL-6, IL-10, IL-2, and IFN-γ levels in COVID-19 patients that was attributed to an activated neutrophils and leucocytes immune function, but the decline in lymphocyte/T Cell response (Gong et al., 2020; Huang et al., 2020b; Liu et al., 2020). Therefore, further studies are warranted to elucidate the nature and cause of these elicited pro-inflammatory cytokines in COVID-19 patients and if it has a relation to SARS-CoV-2 immuno-invasive activity.

### Neuronal/Nervous System Route

SARS-CoV-2 particles were primarily suggested to enter the nerve termini and undergo replication before transportation to the soma and ensuring CNS invasion (Li et al., 2020b). Among the potential SARS-CoV-2 neuroinvasion routes, the olfactory tract serves as an important route for respiratory viruses (Meinhardt et al., 2020; Figure 2). Also, the peripheral nerves namely trigeminal and vagus nerves that innervate distinct parts of the respiratory tract were suggested to be the target of SARS-CoV-2 neuroinvasion (Yavarpour-Bali and Ghasemi-Kasman, 2020). SARS-CoV-2 invades the neural-mucosal interface *via* its transmucosal entry across the nervous assemblies followed by their access to the olfactory tract of the CNS (Meinhardt et al., 2020). Coronaviruses can also invade CNS *via* a synapse-connected route by infecting the peripheral nerve terminals (Dube et al., 2018; Lima et al., 2020). Dube et al. (2018) earlier claimed that human coronavirus (HCoV OC43) might also actively transport through axonal transport by axoplasmic flow and/or may passively diffuse across this channel. SARS-CoV-2 is believed to enter CNS *via* trigeminal nerves that innervate nociceptor cells in the nasal fossa; while the sensory terminal of the trigeminal nerves exits in the conjunctiva (Lima et al., 2020). The finding of SARS-CoV-2 RNA fragment in the ocular discharge of a patient with conjunctivitis (Zhang et al., 2020) further suggested trigeminal nerve-mediated entry of SARS-CoV-2 to CNS.

### Lymphatic-Cerebrospinal Fluid (CSF) Route

The bronchus and trachea tissues comprise a rich and intricate lymphatic network. The olfactory nerve perineural and nasal lymphatic tissue space is suggested to facilitate the CSF drainage by communicating with the channels constituted by ensheathing cells (Figure 2). Of note, endothelial cells in lymphatic networks express CD209L receptor that was claimed to be another receptor facilitating coronavirus invasion (Li et al., 2007). The presence of SARS-CoV-2 nucleocapsid protein in the cells of lymphoid organs affirmed the functioning of the CSF route in the SARS-CoV-2 neuroinvasion (Chen et al., 2020b). These pieces of evidence postulating invasion of CNS by SARS-CoV-2 involve perivascular or lymphatic path as an alternative route (Ylikoski et al., 2020).

### Infected Immune/Lymphatic Cells

Coronaviruses-infected lymphatic/immune cells i.e., T cells, monocytes, and neutrophils were suggested to serve as reservoirs for the virus and were capable to enter and infect the CNS (Iadecola et al., 2020; Figure 2). These immune cells travel to the brain through the meninges and the choroid plexus vasculature (Engelhardt et al., 2017), which could serve as the entry sites for SARS-CoV-2 infected immune cells. As discussed earlier, immune cells also express ACE2 that serves as the molecular receptor for coronaviruses (Lima et al., 2020). Although presently we lack any direct clinical evidence of SARS-CoV-2 invasion of immune cells (Merad and Martin, 2020), immunoreactivity of CD169+ cells for SARS-CoV-2 nucleocapsid protein was seen in the lymph node splenic marginal zone and marginal sinuses (Chen et al., 2020b). Given the fact that CD169+ macrophages amply express ACE-2, makes them a potential target of SARS-CoV-2 neuroinvasion that may further facilitate the entry of infected immune cells to CNS (Park, 2020). Consistent with this, the presence of viral RNA in the macrophages of broncho-alveolar lavage in the COVID-19 patient further highlights the role of infected lymphatic/immune cells in SARS-CoV-2 neuroinvasion (Bost et al., 2020). This evidence postulates that SARS-CoV-2 may infect circulating immune cells and could potentially exploit them to disseminate/invade through the CNS (Figure 2). However, it is still unclear if such presence of SARS-CoV-2 virions/single strand RNA is due to its macrophage invasion or was a result of active phagocytic uptake of the infected cell or SARS-CoV-2 virion (Bost et al., 2020; Merad and Martin, 2020). In contrast, few clinical autopsy reports from COVID-19 patients showed a lack of any infected immune cell infiltration to CNS (Kantonen et al., 2020; Solomon et al., 2020).

The secretion of interferons is the foremost antiviral defense acquired by the immune cells that also stimulate the neighboring immune cells. Coronaviruses can evade the host immune response by producing severe leukopenia and lymphopenia (Wong et al., 2003; Zaki et al., 2012). Earlier investigations on SARS-CoV and MERS-CoV revealed that coronaviruses encode proteins that modulate downstream regulation of TLRs and the JAK-STAT signaling pathway by interacting with their effectors in immune cells. For instance, SARS-CoV and MERS-CoV encoded protein PLpro, inhibits the NF-κB from IκBα dissociation, while, SARSCoV’s PLpro and ORF3b proteins block IRF3 phosphorylation and its nuclear translocation (Devaraj et al., 2007; Signaling et al., 2009). The role of these viral proteins was also implicated in the inhibition of the JAK-STAT pathway (Menachery et al., 2014). In the case of SARS-CoV-2 infection of immune cells, an overall decline in the transcription of antiviral genes was reported due to decreased Type I and III interferons production and an elevated chemokine secretion (Blanco-Melo et al., 2020). Of note, results from *in vivo* and *ex vivo* SARS-CoV-2 experiments affirmed *in vitro* findings and thereby suggested that a decline in the innate antiviral response with instigated hyper-inflammation, could be a potential mechanism of SARS-CoV-2 invasion of immune cells and may contribute to COVID-19 severity (Blanco-Melo et al., 2020). Apart from reducing the T cell number, SARS-CoV-2 also causes effector T cell exhaustion as another mechanism to compromise immune cell function (Diao et al., 2020; Zheng et al., 2020). SARS-CoV-2 exhausted effector T cells as a result show elevated levels of inhibitory receptors *viz*. PD-1, TIM-3, and TIGIT at its surface given the IL-6, IL-10, and TNF-α exposure and declined regulatory T cell function (Chiappelli et al., 2020; Qin et al., 2020).

## Potential Mechanisms of SARS-COV-2 Induced Neurological Injury

As mentioned, SARS-CoV-2 invasion requires ACE2 for S-protein binding followed by its priming by cell proteases TRMPSS2 (Hoffmann et al., 2020). Recent studies also implicated the role of heparan sulfate at the host cell membrane in facilitating the S-protein and ACE2 binding and viral invasion (Clausen et al., 2020; Kalra and Kandimalla, 2021). Co-expression analysis of ACE2 and TMPRSS2 revealed that nasal goblet, ciliated epithelial cells, and oligodendrocytes ubiquitously express both the proteins (Sardu et al., 2020). More specifically, ACE2-TMPRSS2 co-expression in oligodendrocytes could potentiate CNS infiltration as clinical features of acute encephalitis as observed in COVID-19 patients (Hung et al., 2003; Ding et al., 2004). As discussed in the earlier section, coronaviruses can invade the CNS either by a neuronal or humoral/hematogenous route (Figure 2). Therein, early anosmia, i.e., a primary feature of SARS-CoV-2 neuroinvasion that occurs through the olfactory bulb, whereas a retrograde migration of human coronaviruses to olfactory nerve and the CNS *via* nasal epithelium was studied in the murine model (Netland et al., 2008). An earlier study reported an eight-fold increase in the frequency of human coronaviruses infected cells in the CNS that was specifically noticeable in the hippocampus post 1–2 weeks of infection (Chan et al., 2020).

An alternative route of CNS entry for coronaviruses is through the BBB that comprises conditions/factors including endothelins, inflammatory mediators, infected macrophages shipping the virus, or directly infected endothelial cells (Edwards et al., 2000; Paniz-Mondolfi et al., 2020; Sardu et al., 2020). Once the virus reaches the CNS, it starts swift trans-neuronal spread and produces neurotoxicity in infected ACE2-positive neutrons, as validated in transgenic mice models (Netland et al., 2008). Although both SARS-CoV-1 and SARS-CoV-2 bind to ACE2 receptor for host cell entry, recent phylogenetic and virus–receptor binding structural data suggested that SARS-CoV-2 recognizes ACE2 more effectively (Wan et al., 2020; Xu et al., 2020). Therefore, high expression of ACE2 in brain endothelial cells, neurons, and glial cells, makes these neurological cells more prone to SARS-CoV-2 neuroinvasion (Hamming et al., 2004). SARS-CoV-2 recognition of ACE2 may disrupt the delicate balance of ACE-ACE2 cerebrovascular control that could result in incessantly activated ACE signal, severe vasoconstriction, or interrupted cerebral autoregulation. Moreover, SARS-CoV-2 pathogenicity in these tissues was found to elicit IL-1β, -2, -6, -7, -8, -10, -17, INFγ, G-CSF, TNFα, MCP1, and macrophage inflammatory protein 1α (Pedersen and Ho, 2020). The triggered levels of these pro-inflammatory factors produce a “cytokine storm” and are known to be associated with poor clinical outcomes. Besides ARDS, cytokine storm produces severe neurotoxicity by compromising the integrity of the BBB, in absence of direct viral transport or neuroinvasion. These features speculated that acute necrotizing encephalopathy (ANE), may essentially be produced by cytokine-induced neurotoxicity (Ouattara et al., 2011). Therefore, cytokine-induced neurotoxicity in SARS-CoV-2 infected patients may upset neurologic outcomes (Allan and Rothwell, 2001).

Among the different age groups of COVID-19 patients admitted to the hospital, the aforementioned neurological mechanisms were severely deregulated in the elderly population and have exposed their vulnerability (reviewed by COVIDview, 2020; Lekamwasam and Lekamwasam, 2020). These mechanisms of neurological complications underlined the role of disrupted immune function or cytokine-induced neurotoxicity in elderly COVID-19 patients. Koff and Williams (2020) reviewed the consequence of diminished immunity in the aging population and how COVID-19 took advantage of it to exploit it further (Koff and Williams, 2020).

## Emerging Role of Artificial Intelligence and Machine Learning in COVID-19 Diagnostics

During this COVID-19 pandemic, hospitals and other healthcare services have experienced severe crises and are opening up to technologies that can be used in clinical settings as a support system for the frontline healthcare workers in the detection and containment of such diseases. AI is one of those technologies with long–term value. Not only diagnostics but AI is also being employed in hospitals to handle the rapidly increasing load of patients. To manage a similar situation in Boston, Partners HealthCare came up with a hotline service for patients, clinicians, and others with concerns related to COVID-19. The main aim of this initiative was: (i) to identify the class of people with mild symptoms who did not need additional care, to provide them with relevant information and direct them to relevant virtual care options; (ii) to manage the small high-risk patients by linking them to testing sites, newly created respiratory illness clinics, or emergency department of hospitals in case necessary. This initiative was further expanded in collaboration with St. Joseph Health system in Seattle and Microsoft, which served more than 40,000 patients in the 1st week itself. In line with this, smart AI bots are also being developed as chatbots to manage the increasing needs of patients and clinicians. Moving a step ahead, a group of researchers at MIT has trained an AI model that can distinguish asymptomatic people from healthy individuals through their forced-cough recordings. The model was able to accurately detect 98.5% of COVID-19 positive people, which included 100% cases of coughs submitted by asymptomatic patients (Laguarta et al., 2020). Though still in its infancy, such technology can be used as a pre-screening tool in various situations. Another group has reported an AI-based screening model for early detection of COVID-19 using the routinely collected healthcare data (laboratory tests, blood gas measurements, and vital signs) that typically become available within the first hour of presentation to any hospital with regular laboratory infrastructure (Soltan et al., 2020). During the 2 weeks of testing phase at the John Radcliffe Hospital in Oxford and the Horton General Hospital in Banbury, this AI model could correctly predict the COVID-19 status of 92.3% of patients admitted to the emergency departments. So, as an alternative to the swab test that takes typically a day’s time for results, this AI screening maintained the flow through the hospital by confidently predicting the negative COVID-19 cases. Several governments and hospitals across the globe are also using AI-powered sensors to identify suspected patients. Of note, physician-researchers at Brigham and Women’s Hospital and Massachusetts General Hospital are trying to make use of intelligent robots (developed at Boston Dynamics and MIT) to monitor vital signs in COVID-19 patients and deliver their medications (Wittbold et al., 2020). This will assure the least human contact with infected patients and thus control disease transmission.

Scaling AI for better explainability and transparency of imaging for diagnostics is another major direction to improve upon. An AI algorithm has also been constituted that integrates a spectrum of chest CT imaging features with clinical symptoms, exposure history, and laboratory testing to rapidly diagnose COVID-19 positive patients. The trained model was able to achieve an Area under the ROC Curve (AUC) of 0.92, indicating a sensitivity comparable to that of a senior thoracic radiologist. It was also able to detect 17 out of 25 COVID-19 positive patients, who were reported as negative by the radiologists (Mei et al., 2020). Jin et al. (2020) have also reported a similar model that makes use of chest CTs for COVID-19 detection. In China, an AI-driven CT scan interpreter has been installed in Zhongnan Hospital that helps in the diagnosis of COVID-19 when radiologists are not available (Wittbold et al., 2020). Furthermore, this technology can have direct implications in the detection of post-COVID-19 neurological complications as well. For example, AI could have been efficiently used in the study to systematically characterize neurological symptoms in COVID-19 infected people that involved neuroimaging of about 108 patients from multiple institutions in Italy (Mahammedi et al., 2020). AI thus holds the promising potential to develop into a mainstream diagnostic for fast and efficient detection of various diseases.

Although AI seems to be a promising solution to our growing healthcare needs, it needs to be executed with human clinical expert decision-making at appropriate levels to ensure high quality and safe delivery of AI outcomes. AI can only be an aid but cannot be a replacement for human clinical reasoning and decision making.

## Conclusion

Emerging clinical data revealed that neurologic manifestations are frequent in elderly and severely sick COVID-19 patients that significantly raised their mortality. Also, existing comorbidities in COVID-19 patients can further contribute to the neurological complications and impact the clinical outcome. Existing neurological conditions, neurodegenerative ailments, and inflammation in the elderly population were found to worsen the clinical outcome in COVID-19 patients. Although emerging clinical evidence underlined the role of neuroinvasion, neuroinflammation, immunopathogenesis, and hypoxemia in the development of CNS manifestations, the molecular mechanism of COVID-19 neurotoxicity is not yet completely known. Therefore, direct involvement of the above events during or post-SARS-CoV-2 infection is unclear to assess their exact clinical outcomes in elderly COVID-19 patients. Given the diverse and complex clinical signatures of COVID-19 affecting multiple cross-histological functions, it warrants more concerted efforts to distinctly characterize the molecular events involved in its pathogenesis, more importantly in elderly patients. Clinical data revealing SARS-CoV-2 presence in the brain postulated neuroinvasion theory, therefore, direct contact of SARS-CoV-2 with the nervous system is clinically relevant. However, the lack of SARS-CoV-2 virion/mRNA in CSF in the majority of the COVID-19 cases hinted at an alternative viral gateway. Also, in COVID-19 patients, SARS-CoV-2-induced inflammation and immune response appeared to further exacerbate the neurologic complications. Given the fact that COVID-19 infection severity has a direct link with the extent of inflammation, the possibility of deregulated immune function in neurotoxicity cannot be excluded. Incidences of “cytokine storm” in SARS-CoV-2 infected patients are seen to greatly contribute to neurological complications, more often in elderly patients. Therefore, in the COVID-19 patients, virus-induced inflammation is suggested to play a key role in potentiating neurological complications. However, it requires further investigation at the molecular and systemic level to precisely define the pathophysiological relevance of these events in neurological complications.

SARS-CoV-2 infected elderly patients are at higher risk of neurological complications. These complications beyond producing acute illness can also exert prolong impact on the functioning of the nervous system. These facts made aged COVID-19 patient’s health management more complex, even greater for those having preexisting comorbidities. Careful assessment of these features in admitted elderly COVID-19 patients with the help of advanced AI and machine learning can make COVID-19 diagnostics more efficient and could also save lives. Conclusively, COVID-19 associated neurological complications are a serious health concern and require more concerted investigative efforts to understand and intervene in their progression in elderly patients.

## Author Contributions

RSK, JKD, and RK conceived the idea. RSK, JKD, AM, VK, BS, SDa, SDe, and RK wrote the manuscript. RSK drafted the manuscript figures. RSK, JKD, BS, and RK supervised and critically revised the study. All authors contributed to the article and approved the submitted version.

## Conflict of Interest

The authors declare that the research was conducted in the absence of any commercial or financial relationships that could be construed as a potential conflict of interest.
